# Genital Peculiarities of the Negro

**Published:** 1903-03

**Authors:** 


### EDITORIAL NOTES AND COMMENTS.

The Business office of THE JOURNAL-RECORD is No. 64 Marietta Street.

The Editorial office is 318-319-320 The Prudential.

Address all Business communications to Dr. M. B. Hutchins, Mgr.

Make remittances payable to THE ATLANTA JOURNAL-RECORD OF MEDICINE.

On matters pertaining to the Editorial and Original Communications address Dr. Bernard Wolff, Atlanta.

Reprints of original articles will be furnished at cost price. Requests for the same should always be made *on the manuscript*.

We will present, post-paid, on request, to each contributor of an original article, twenty (20) marked copies of THE JOURNAL-RECORD containing such article.

### GENITAL PECULIARITIES OF THE NEGRO.

AMONG the many points of difference that exist in the physical make-up of the white and black types of mankind, there are perhaps none more striking than those presented by the reproductive organs, especially those of the male.

As the sexual function is so intimately associated with the lower sensual or emotional nature, and as this among negroes is the dominant note, it is natural to look for excessive development in the organs connected with it. And as a matter of fact this hypertrophy notoriously exists. Among negroes the virile organ attains its maximum development often reaching massive proportions. Even before the age of puberty it is frequently noted to exceed in size that of an average adult white man's. This is a racial characteristic and can not be explained from the early establishment of the physiological function of the organ and its employment in this capacity at what in other races would be considered an asexual age. This generousness of dimension is usually increased by elongation of the foreskin, which is the rule in the negro rather than the exception as among white men, and may not be an unimportant factor in arousing by preserving sensitiveness of the glans that excessive *libido sexualis* which is the negro's birthright and conspicuous trait. To offset this penile excess, the testicles are, as a rule, smaller than among white men; the scrotum is also shorter and less voluminous and is frequently retracted after the manner of a dog's. The shortness of the sack offering support to the testicles within may in a measure explain the rarity of varicocele in the negro—a fact frequently noted and commented upon by those in the South who are concerned with genito-urinary work, for which such abundant clinical material is offered by negroes.

Inversion, perversion or other abnormality of the sexual sense is very infrequent among negroes if the sadistic tendencies toward mutilation of his victim shown sometimes by the black rapist be excepted. It is natural to suppose that such perversions of a function which is physiologically almost perfect, and which remains so to extreme age in negroes, would be rare, as it is usually found among those higher up in the scale of humanity who have become *blasé* and are forced to resort to artificial means of gratifying a weakened or paralytic function, or who in this respect are defective by reason of a neuropathic heredity. These circumstances and factors do not obtain among the blacks.

Among women sexual differences are much less marked. There occurs with some frequency a certain atrophic condition of the external genital organs in which the labia are much flattened and thinned, approaching in type that offered by the female anthropoid ape, hepale, lemur and other pithecoid animals. Enlargement of the clitoris is no oftener seen among colored women than white, and when found is usually due to disease. The mammary glands of negresses are usually longer than among white women, more cylindrical, with somewhat more projecting nipples.

The genital characteristics coupled with utter contempt and cynical disbelief in the existence of chastity, together with his stallion-like passion and entire willingness to run any risk and brave any peril for the gratification of his frenetic lust, render the negro the menace of the rural South. He is the terror that stalks abroad—the very thought of whom sends a chill of sickening fear to the heart of the woman in the lonely farm-house and drives men to deeds of savagery. How much longer will the South have to bear this burden until deliverance comes?

Can not those who so freely criticise the attitude of the Southern people toward this unmerited infliction take it to their own bosoms? They are welcome to him, and if he would raise the old cry of, "we are coming, Father Abraham, three hundred thousand strong," none would welcome the exodus more than the Southern people, although it would be pretty rough on Abraham.

